# Spike protein evolution in the SARS-CoV-2 Delta variant of concern: a case series from Northern Lombardy

**DOI:** 10.1080/22221751.2021.1994356

**Published:** 2021-11-01

**Authors:** Andreina Baj, Federica Novazzi, Francesca Drago Ferrante, Angelo Genoni, Elena Tettamanzi, Giuseppe Catanoso, Daniela Dalla Gasperina, Francesco Dentali, Daniele Focosi, Fabrizio Maggi

**Affiliations:** a Laboratory of Microbiology, ASST Sette Laghi, Varese, Italy; b Department of Medicine and Surgery, University of Insubria, Varese, Italy; c ATS Insubria, Varese, Italy; d North-Western Tuscany Blood Bank, Pisa University Hospital, Pisa, Italy

**Keywords:** SARS-CoV-2, Delta, S71F, T250I, T572I, K854N, G769V, C1238F

## Abstract

The SARS-CoV-2 variant of concern (VOC) “Delta” is currently defined by PANGOLIN as a cluster of 33 different AY sublineages. Delta (in particular B.1.617.2) is largely and rapidly replacing the Alpha VOC as the dominant clade in most countries. To date, variations in the Spike protein of the Delta VOC have largely been limited. We report here the results of a genomic surveillance programme from Northern Italy. We identified several Delta sublineages harbouring mutations previously reported in GISAID at extremely low frequencies and in different combinations. Two patients (one of them vaccinated) tested positive for a Delta sublineage harbouring S71F, T250I, T572I and K854N. More patients tested positive for G769 V plus C1248F, A352S, and R158G and C1248F, respectively. Genomic surveillance of Delta variants should be encouraged to anticipate immune escape and deploy countermeasures.

## Introduction

SARS-CoV-2 is an RNA virus with a relatively stable genome, largely thanks to its proofreading exonuclease. Nevertheless, the aggressive pandemic has led to massive replication events worldwide, and around 800 SARS-CoV-2 sublineages have been reported to date. The most challenging sublineages (in terms of increased transmissivity, morbidity, or immune escape) have been named “variants of concern” (VOC). The WHO has recently dubbed VOCs with a simplified Greek alphabet nomenclature, easy to recall than former phylogenies provided by GISAID, PANGOLIN, NextStrain, and Public Health England (PHE). While in the first semester of 2020 Alpha (a.k.a. B.1.1.7 or “UK variant”) was the predominant VOC, the second semester of 2021, after massive vaccination campaigns, saw the growth of Delta (a.k.a. “Indian variant”). The Delta VOC, identified in October 2020 in India, consists of 41 different sublineages sharing additional T19R, del157/158, T478K, and D950N in Spike protein and I82T in M protein compared to B.1. The beta-loop-beta receptor-binding motif (RBM) adopts an altered but stable conformation causing separation in some of the antibody binding epitopes [1]. Interestingly, Spike mutations in Delta variants distribute in a similar way to those of other VOCs, clustering in the N-terminal domain (NTD) targeted by most neutralizing antibodies, or in the peripheral ACE2 receptor-binding domain (RBD). This pattern suggests that the virus accumulates mutations in order to reduce or avoid recognition by antibodies but maintaining or increasing binding to ACE2[1, 2]. Is therefore important to track the emergence of new spike mutations potentially affecting antigenic structure.

According to Italian Ministry of Health guidelines, we started a genomic surveillance programme in the Lombardy Region in Northern Italy.

## Materials and methods

Between June 2021 and July 2021, 2,622 (8.8%) out of 29,666 nasopharyngeal swabs (NPS) tested positive for SARS-CoV-2 RNA at our laboratory by using several real-time reverse transcriptase (RT)-PCR platforms. 815 of these 2,622 positive samples underwent sequencing for characterizing the infecting SARS CoV-2 variant, according to the surveillance programme of the Italian National Institute of Health and Ministry of Health. Of these 815, 97 samples were sequenced by Sanger for the entire Spike gene or by next-generation whole-genome sequencing (NGS) as previously reported [3]. Briefly, Sanger protocol was performed on RT–PCR fragments covering the complete Spike gene of SARS-CoV-2, and directly using the Big Dye Terminator cycle-sequencing kit (Applied Biosystems). Cycle sequencing was carried out with an automatic DNA sequencer (ABI model 373; Applied Biosystems) and performed in both orientations for confirmation. Nucleotide Spike gene sequences were aligned with previously published sequences. Complete SARS-CoV-2 genome was obtained by using the amplicon-based SARS-CoV-2 FLEX NGS panel on Miseq platform (Illumina Inc., San Diego, USA) and the SOPHiA DDM® software (Sophia Genetics, Inc, Boston, USA) for sequence analysis. Frequencies of each mutation were calculated among 3,578,384 SARS-CoV-2 genome sequences deposited in GISAID as of September 21, 2021 using the Oubtreak.info web portal (https://outbreak.info/situation-reports).

## Results

Out of the 97 Spike or full-genome sequences, 76 (78%) harboured the Delta variant. Among them, we describe here 5 patients who harboured sequences with additional Spike mutations very rarely reported (or unreported in such combination) in the GISAID database. Table 1 summarizes details for each case.

**Table 1.** Summary of main details for the 4 Delta sublineages reported in the current survey.

sublineage	case (gender/age)	GISAID entry	extra mutation in S protein	overall frequency as of September 21, 2021	frequency in Delta sublineage as of September 21, 2021
#1	F/26 M/65	EPI_ISL_3232432 EPI_ISL_3232507	S71F	2,003 sequences, the highest frequency (2%) occurring in the B.1.605 strain from the USA	B.1.617.2: 102 out of 522,766 AY.5: 25 out of 28,097 AY.7.2: 1 out of 2,002 AY.4: 110 out of 390,000 AY.3: 6 out of 33,823 AY.20: 2 out of 12,423 AY.1: 1 out of 12,423 AY.6: 1 out of 12,109 AY.25: 4 out of 53,493 AY.9: 2 out of 30,948 AY.12: 3 out of 49,152
			T250I	6,194 sequences, the highest frequency (1%) occurring in B.1.617.2	B.1.617.2: 4,862 out of 522,766 AY.11: 6 out of 984 AY.10: 32 out of 7,165 AY.21: 4 out of 952 < 0.5% in AY.7.1, AY.25, AY.22, AY.19, AY.12, AY.4, AY.13, AY.24, AY.5, AY.7.2, AY.23, AY.1, AY.20, AY.14, AY.3, AY.6, AY.9
			T572I	12,017 sequences, being a hallmark mutation of the uncommon B.1.177.88 and B.1.1486 lineages, and partly of the B.1.284 lineage	B.1.617.2: 2,058 out of 522,766 AY.16: 30 out of 1,629 AY.5.1: 2 out of 233 < 0.5% in AY.7.2, AY.7.1, AY.4, AY.12, AY.25, AY.21, AY.10, AY.23, AY.15, AY.7, AY.24, AY.13, AY.3, AY.5, AY.20, AY.2, AY.3.1, AY.9, AY.6
			K854N	2,768 sequences, being the hallmark mutation of the uncommon B.1.1.384 lineage, and partly of the B.1.623 lineage from the USA	B.1.617.2: 348 out of 522,766 AY.22: 20 out of 433 AY.4: 1,054 out of 390,000 AY.10: 10 out of 7,165 AY.15: 3 out of 2,868A AY.19: 1 out of 1,335 AY.7.2: 1 out of 2,002 AY.9: 15 out of 30,948 AY.13: 1 out of 2,956 <0.5% in AY.14, AY.12, AY.5, AY.25, AY.6, AY.20
#2	F/44	EPI_ISL_3232508	G769V	15,725 sequences, being the hallmark mutation of lineages B.1.1.91, R.1, B.1.177.78, B.1.1.123, and B.1.1.421	AY.1: 3 out of 1237 AY.24: 8 out of 4,350 AY.19: 2 out of 1,235 AY.16: 2 out of 1,629 AY.4: 235 out of 390,000 B.1.617.2: 297 out of 522,766 < 0.5% in AY.9, AY.7.1, AY.12, AY.3, AY.10, AY.5, AY.6, AY.3.1, AY.7, AY.23, AY.25, AY.20, AY.14
			C1248F	989 sequences, with the highest (5%) prevalence in B.1.264.1 lineage	AY.21: 2 out of 952 AY.20: 21 out of 12,423 AY.1: 1 out of 1,237 AY.4: 275 out of 390,000 AY.24: 3 out of 4530 B.1.617.2: 272 out of 522,766 < 0.5% in AY.12, AY.23, AY.7, AY.7.1, AY.10, AY.3, AY.25, AY.5
#3	M/23	EPI_ISL_3239899	A352S	989 sequences, being the hallmark mutation of lineage AH.3 (98% prevalence)	B.1.617.2: 43 out of 522,766 AY.19: 1 out of 1,335 AY.14: 4 out of 6,467 AY.15: 1 out of 2,868 AY.10: 2 out of 7,165 AY.4: 95 out of 390,000 AY.6: 2 out of 12,109 AY.23: 1 out of 9,258 AY.20: 1 out of 12,423 AY.3: 2 out of 33,823 AY.12: 1 out of 49,152 AY.25: 1 out of 53,493
#4	M/45	EPI_ISL_3239897	R158G	1,154 sequences	AY.3.1: 116 out of 4,161 AY.25: 326 out of 53,493 AY.13: 10 out of 2,956 AY.22: 1 out of 433 AY.3: 59 out of 33,823 AY.1: 2 out of 1,237 AY.2: 3 out of 2,157 AY.10: 9 out of 7,165 B.1.617.2: 218 out of 522,766 < 0.5% in AY.23, AY.4, AY.8, AY.14, AY.16, AY.5, AY.12, AY.71, AY.9
			C1248F	989 sequences	B.1.617.2: 272 out of 522,766 AY.21: 2 out of 952 AY.20: 21 out of 12,423 AY.1: 1 out of 1,237 < 0.5 in AY.4, AY.24, AY.12, AY.23, AY.7, AY.7.1, AY.10, AY.3, AY.25, AY.5

A first sublineage was identified in two patients, a 65-years old male and a 26-years old female nurse. The male patient was an international trader and had a history of 2 former NPS positivities, the first in Italy in November 2020 and the second in Kenya (where he had moved since April 2021) in May 2021, both during voluntary screenings while asymptomatic. After a 15-days quarantine in Kenya, he fled to South Africa, where he fell ill with severe pneumonia and tested positive for a third time on June 7, 2021. He was repatriated with a dedicated flight and admitted to the hospital on June 15, 2021. His anti-S IgG serologies (DiaSorin, Saluggia, Italy) were negative on February 2021 and on June 17. The patient fully recovered, his NPS was negative on June 25 and was discharged the following day. The female patient was a nurse at the COVID hospital who was quarantined before having contact with the male trader. She had been fully vaccinated with BNT162b2 since January 2021, and follow-up anti-S IgG serologies (DiaSorin) were positive on March 1 (367 AU/ml) and June 18 (752 AU/ml). On June 10 she had short contact with her father, who had tested positive for a SARS-CoV-2 antigen assay the day before and had got the infection from a Tunisian immigrant: she fell ill on June 13 and tested positive at PCR on June 14. Her 20-years old brother, who lived with the father, fell ill on June 10, tested positive on June 14, and developed severe pneumonia: none of the nurse colleagues tested positive at surveillance NPSs. The patient recovered and a follow-up NPS on July 1 was negative. Both the trader and the nurse harboured identical B.1.617.2 sequences, additionally harbouring S71F, T250I, T572I, and K854N [4].

A second sublineage was identified in a 44-years old immunocompetent female whose NPS tested positive (cycle thresholds 22 for gene E and 23 for gene N2) on June 22, after strict contact with her mother, which was a confirmed COVID-19 case admitted to the hospital two days before. The patient had received 2 doses of Vaxzevria® on March 15 and June 1, 2021. She developed anosmia, ageusia, mild fever, fatigue, and dyspnoea. The Spike sequence harboured G769 V and C1248F as additional mutations. These mutations were previously unreported in Delta VOCs.

A third sublineage was reported in a 23 years-old male who tested positive on July 13 (cycle threshold 18 for gene E and N2). The sequence confirmed Delta variant additionally harbouring A352S.

A fourth sublineage was reported in an unvaccinated, immunocompetent 45 years-old male returning from Barcelona by plane on June 1. He developed myalgia and mild fever, and tested positive to NPS on June 4 and 9; a follow-up NPS was negative on June 16. His 74-years old immunocompetent father (who had received a single Vaxzevria® dose) tested positive on June 12, developed mild respiratory symptoms, and tested negative on June 23. His 84-years old immunocompetent mother (who had received 2 doses of Comirnaty® on March 31 and April 21), first tested positive on July 5. The sequence confirmed Delta variant additionally harbouring R158G and C1248F.

## Discussion

The Delta VOC is becoming the predominant SARS-CoV-2 strain worldwide, largely replacing the Alpha (B.1.1.7) VOC thanks to higher transmissivity.

**Figure 1.**
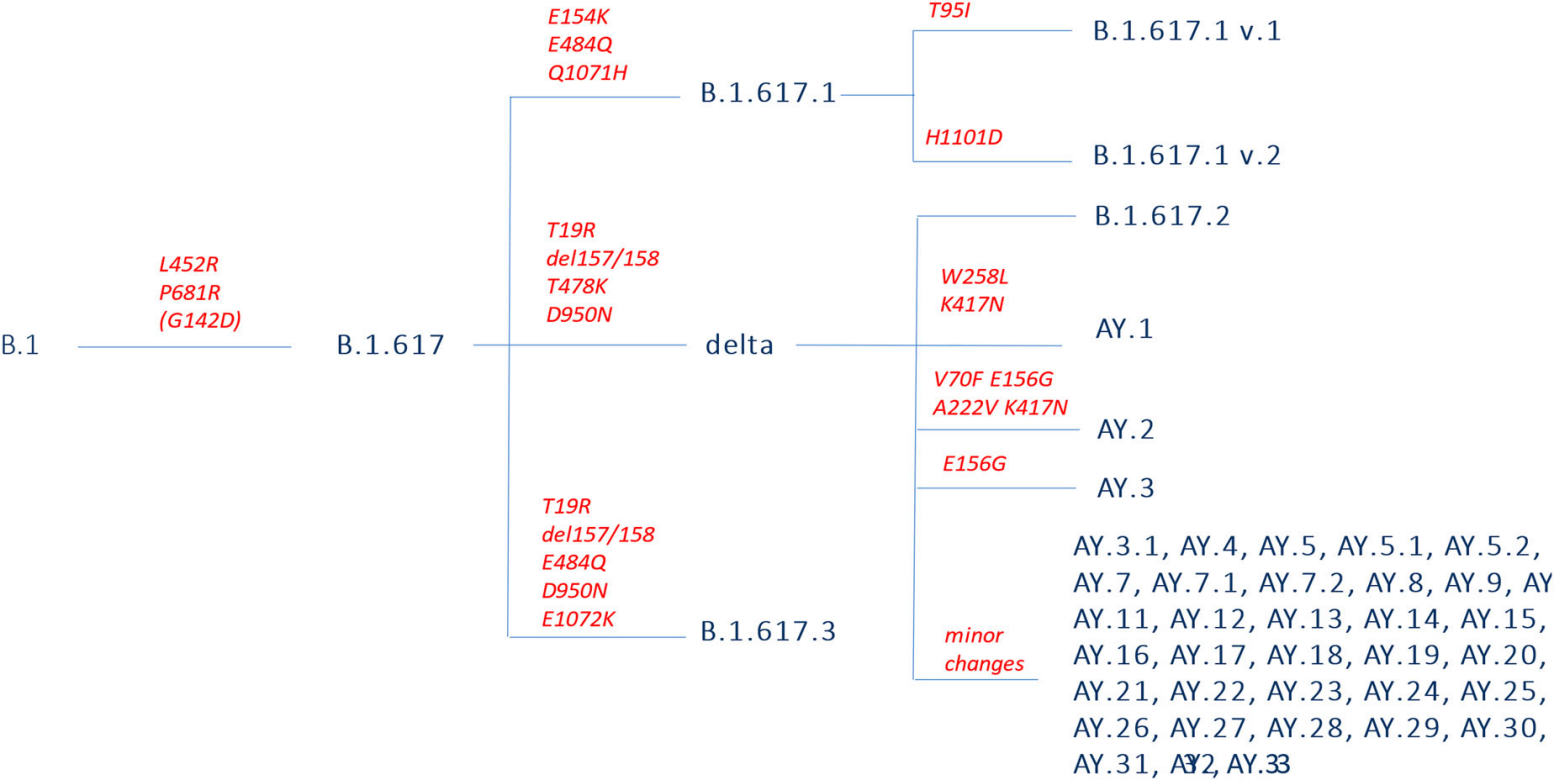
Simplified linear representation of the delta VOC subclades currently reported by WHO.

The differences between the main Delta sublineages are summarized in Figure 1. B.1.617.2 (a.k.a. VUI-21APR-02 by PHE) is the reference strain of Delta, the first to be reported and largely the most prevalent. Del157/158 in the NTD of Spike leads to immune evasion through antibody escape [5]. B.1.617 entered 2 out of 8 cell lines tested (including Calu-3 lung cell [6]) with increased efficiency. K77T and T95I (typical of B.1.617 v.1), L216F, A222V, and G1124V have also been rarely reported. B.1.617.2 is resistant to bamlanivimab [5, 6] and moderately evades convalescent or BNT162b2-elicited sera [6, 7], which is similar in magnitude to the loss of sensitivity conferred by L452R or E484Q alone. Furthermore, P681R mutation significantly augments syncytium formation in Calu-3 cells [7] and hamsters [8] compared to the B.1.617.1 Spike protein, potentially contributing to increased pathogenesis observed in hamsters and infection growth rates observed in humans [8]. In hamsters, higher shedding of subgenomic RNA has been reported for 14 days, and moderate lung pathology has been reported in 40% of infected animals [9]. Delta can cause vaccine breakthrough events in humans [10] and has been reported to infect Asiatic lions (*Panthera leo persica*) in Arignar Anna Zoological Park, Chennai, Tamil Nadu, India [11]. Analysis of an outbreak involving 167 cases from China showed that the time interval between exposure to first positive PCR was shorter (4 vs 6 days), the initial viral load 1260-folds higher and more infectious (80% vs. 19% harbouring > 6 × 10^5 viral copies/ml) than in 19A/19B infection: some minor intra-host single nucleotide variants (iSNVs) could be transmitted between hosts and finally fixed in the virus population during the outbreak. The minor iSNVs transmission between donor-recipient contribute at least 4 of 31 substitutions identified in the outbreak suggesting some iSNVs more likely to arise and reach fixation when the virus spread rapidly [12]. Neutralization by CCP, BNT162b2, mRNA-1273 and Ad26.COV2.S-elicited antibodies is reduced by 3–5 folds, while REGN10933 efficacy is reduced by 12 folds compared to wild-type (having only a minor effect on the activity of the REGN-COV2 cocktail) [13].

AY.2 additionally harbours V70F (unique among delta lineages), A222V (shared with AY.9, AY.10, AY.11, AY.19 and AY.24) and K417N (as for AY.1) K356N and V1228L have also been rarely reported. AY.3 additionally harbours E156G. AY.3.1 never harbours T95I nor A222V, and harbours F157C and R158G at about 10% frequencies.

As expected for a strain with massive circulation, quasi-species are arising across the world. Subclades for Alpha and other VOCs have been relatively uncommon (e.g. Q.1 to Q.8 for Alpha, B.1.351.1. to B.1.351.4 for Beta, and P.1.1 to P.1.11 for Gamma), but Delta moves from as a mix of 41 sublineages, making further diversification easier. Our analysis derives several limitations from GISAID: deposited sequences are a sample of the total number of cases and may not represent the true prevalence of the mutations in the population, and case numbers for any given lineage/mutation can be significantly affected by overall case numbers and rates of genomic sequencing at any given location.

We have reported here the heterogeneity of the Delta VOCs in a small series from a single Italian laboratory, which suggests the real heterogeneity of Delta is far higher than previously supposed. The Delta VOC has reduced to *in vitro* neutralization by convalescent plasma from previous waves [7, 13–16], monoclonal antibodies (such as bamlanivimab) [2, 6, 7, 14, 17], casirivimab [13], regdanvimab [18] and vaccines such as BNT162b2 [6–8, 2, 13, 19–23], mRNA-1273 [13, 14, 19, 22–24], AZD1222 [2, 13, 25, 26], Ad26.COV2.S [27], CoronaVac [15, 28] and BBV152 [16, 29]. Given such concerns, genomic surveillance is mandatory to intercept (and eventually contain) Delta-derived variants earlier. Furthermore, the samples submitted for sequencing are often not a random sample of all cases, which skews the estimated prevalence of a lineage in a location.

## Abbreviations

**Abbreviations:** VOC: variant of concern.
